# Bone density, fractures and the associated factors in iranian children and adolescent with Osteogenesis Imperfecta

**DOI:** 10.1186/s12887-020-02491-1

**Published:** 2021-01-14

**Authors:** Pooran Mohsenzade, Anis Amirhakimi, Naser Honar, Forough Saki, Gholam Hossein Ranjbar Omrani, Mohammadhosein Dabbaghmanesh

**Affiliations:** 1 Shiraz University of Medical Sciences, Shiraz, Iran; 2 Gastroentrology Research Center, Neonatology Research Center, Shiraz University of Medical Sciences, Shiraz, Iran; 3 Shiraz Endocrinology and Metabolism Research Center, Shiraz University of Medical Sciences, P.O. Box: 71345-1744, Shiraz, Iran

**Keywords:** Children, Osteogenesis Imperfecta, Vitamin D, BMD, Iran

## Abstract

**Backround:**

Osteogenesis imperfecta(OI) is a frequent bone fragility disorder in children. The purpose of this study was to assess the BMD and Vitamin D level in children with OI in southern Iran.

**Method:**

This case-control study was conducted on 23 children, clinically diagnosed as osteogenesis imperfecta and 23 age- and gender-matched healthy controls. Demographic and anthropometric data, biochemical parameters, puberty, sun exposure and physical activity were assessed. Bone mineral density (BMD) was measured by Dual-energy X-ray absorptiometry (DXA). Data analysis was done by SPSS22.

**Results:**

Forty-three point four percent of OI patients and fifty-six point five percent of control group had vitamin D deficiency (*P* = 0.376). Thirteen OI patients (56%) had low bone mass for chronological age in lumbar area (*P* < 0.001). Fracture episodes during treatment was significantly influenced by time of Pamidronate start, courses of Pamidronate injection, puberty and sun exposure (*P* values = 0.015, 0.030, 0.044 and 0.032, respectively). Fracture episodes during treatment had significantly increased in patients who had received Pamidronate more than 3 years compared with those received less than 3 years(*P* values = 0.047).

**Conclusions:**

This study showed that vitamin D deficiency is prevalent amongst OI children in southern Iran. More than half of the OI children had low bone mass for chronological age in lumbar area, despite receiving bisphosphonate therapy. The present results revealed that early initiation of Pamidronate and number of Pamidronate courses are associated with lower fracture rate. However, treatment period more than 3 years can have adverse effect on fracture rates.

## Mini abstract

This study showed that vitamin-D deficiency is prevalent amongst OI children in southern Iran despite supplemental vitamin-D. 56% of patients had low bone mass despite receiving bisphosphonate therapy. Early initiation and number of Pamidronate courses are associated with lower fracture rate. Treatment longer than 3 years can aggravate fractures.

## Background

Osteogenesis imperfecta(OI), or brittle bone disease, is one of the most common primary bone fragility disorder in children and adolescents. It is due to a mutation in one of the two genes coding for collagen type I, the genes associated with the modification of collagen type 1 and some transcription factors are related with osteoblast differentiation [1, 2]. Its prevalence was reported as 1 in 15–20,000 births [1]. Diagnosis is based on history, clinical findings and radiographic findings of repeated old and new fractures which showed no criteria of other metabolic bone disease. Radiographs was used to ruled out other metabolic bone disease such as rickets. Its clinical manifestations are classified as skeletal and extra-skeletal. Skeletal features include bowing deformities of long bones, macrocephaly, fracture due to mild trauma, face malformation, chest wall deformities and short stature. Extra-skeletal manifestations consist in blue sclera, dentinogenesis imperfecta, and hearing impairment [2, 3]. OI has been categorized, according to clinical presentation into four types(types I– IV), based on Sillence criteria [4]. This criteria was introduced in 1979 according to clinical and radiological findings and mode of inheritance, prior to the availability of molecular genetic analyses. Type I is mild and the most frequent type without bone deformity and short stature. it`s inheritance is autosomal-dominant and these patients may have blue sclera. OI type II is the pre- or perinatal lethal form because of multiple fractures, osteopenia and pulmonary hypoplasia. OI type III is the most severe type in children surviving the neonatal period, suffer several peripheral and vertebral fractures. At last, OI type IV is an overlapping phenotype of OI types I and III with variable clinical presentation. Clinical OI types II, III, and IV have either autosomal or recessive inheritance.

Unfortunately, treatment of OI patients is limited to supportive care and there is no cure for them. According to the disease severity, degree of impairment and age of the individual, mode of therapy is varied and individualized. These therapies include orthopedic management e.g. surgical intervention or bracing of lower limbs, physical and occupational therapy, and drugs including bisphosphonates, growth hormone, calcitonin, parathyroid hormone, sodium fluoride, vitamins. Bisphosphonates are currently the most useful pharmacologic therapy. Nearly all the previous reports evaluating BMD in patients with osteogenesis imperfecta independently reported a significant increase in BMD after treatment with either oral or IV. However, it is difficult to compare the results of these trials because they had studied different populations (adults versus children; because children cannot be compared with adults due to high bone turnover during growth years with open epiphyses). Moreover, although OI patients are taking Bisphosphonates like Pamidronate to increase bone density and reduce fractures [5], the patients suffer primary osteoporosis, most observed in lumbar area due to intrinsic bone disease [6, 7]. Studies from around the globe showed different prevalence rates of osteopenia in patients with OI; hence, it is important to evaluate bone density and fracture risk in OI patients to prevent future fractures and disabilities and improve their quality of life. Also, understanding the prevalence of osteoporosis and its related factors in children with OI can help the pediatricians to do a more accurate management of OI patients [8–10].

In addition, vitamin D plays an essential role in obtaining adequate bone mineralization, and also has a positive effect on muscles, which can indirectly have a favorable influence on bone [6]. Low levels of serum 25-hydroxyvitamin D(25OHD) in many children and adolescents with OI has been shown in prior studies [6, 9, 11, 12].

The purpose of this study was to evaluate Vitamin D deficiency, bone mineral density, fracture rate and its associated factors amongst children and adolescents with Osteogenesis Imperfecta in a case-control study in southern Iran.

## Methods

### Study design

This study case was conducted on 23 patients in Shiraz, a city in southern Iran, clinically diagnosed as Osteogenesis Imperfecta who were referred to the pediatric endocrinology ward of Namazi hospital affiliated with Shiraz University of Medical Sciences (SUMS), for receiving intravenous Pamidronate, during spring and summer of 2018. Inclusion criteria were clinical diagnosis of OI type I, III, or IV which had diagnosed and followed in pediatric endocrinology clinic and age of 1–18 years. Exclusion criteria were secondary causes of osteoporosis including any systemic inflammatory or autoimmune disease, liver or kidney disease, diabetes mellitus, and any medical history of malignancy. Demographic data and detailed medical history (fracture rate, time of the first fracture, history of intrauterine fracture and treatment period) was obtained from each patient. At the time of admission, all patients were taking supplemental calcium and vitamin D (500 mg calcium carbonate and 200 IU vitamin D per day). Twenty-three healthy age- and gender-matched children were recruited from the local schools in Shiraz through an age/gender-stratified randomly selected sampling method as the control group.

### Body size, pubertal status, physical activity and sun exposure

A trained physician measured weight, height or length and pubertal stage of the participants. The height was recorded, using a standard wall-mounted meter [13], while the children were standing without shoes and it was rounded to the nearest 0.5 cm. Infants and children unable to stand were measured in the supine position. Weight was measured with a standard scale (Seca, Germany) while the children wore a light clothes without shoes and it was rounded to the nearest 0.1 kg. Body mass index (BMI) was calculated using the standard formula: 

$$ \mathrm{BMI}\ \left(\mathrm{kg}/\mathrm{m}2\right)=\mathrm{weight}\ \left(\mathrm{kg}\right)/\left[\mathrm{height}\ \left(\mathrm{m}\right)\right]2 $$

BMI was converted to age- and gender-specific percentile according to reference data published by the Centers for Disease Control and Prevention [14].

Evaluation of puberty was assessed through the Tanner standard staging system by examination of a pediatric endocrinologist [15]. Participants were categorized as pre pubertal (Tanner 1), early pubertal (Tanner 2 and 3), and late pubertal (Tanner 4 and 5). According to the last recommendation of the American College of Sports Medicine, physical activity was classified into sufficient (3 days of physical activity per week) and insufficient groups (less than 3 days per week) [16]. It includes walking, riding bicycle, playing aerobic exercise such as football. Furthermore, we categorized the patients based on their subjective report of daily sun exposure (during 10 a.m. – 4 p.m.) into three groups: (1) low exposure, less than 15 min/day; (2) medium exposure,15–30 min/day; and (3) high exposure, more than 30 min sun exposure daily [17].

### Biochemical parameters

All the blood samples were taken by an expert technician after 8–12 h of fasting (before starting Pamidronate) and assessed in Shiraz Endocrinology and Metabolism Research Center. Serum calcium (Ca), Phosphorous (P), and Alkaline phosphatase (ALP) were checked using colorimetric assay with an auto analyzer (Bio system SA, Barcelona, Spain). Serum concentration of 25-hydroxy vitamin D(25OHD) was assessed with a high performance liquid chromatography (Young Lee 9100, South Korea) in ng/ml. The intra-and inter-assay coefficients of variation were 8.2 and 8.4%, respectively. Based on the last guideline published by the Endocrine Society for Clinical Practice, serum concentration of 25OHD less than 20 ng/ml was defined as vitamin D deficiency [15].

### Bone mineral density

Bone mineral density measurement of individuals was done by Hologic system DXA (Discovery QDR, USA). The standard data of Hologic system DXA (Discovery QDR, USA) which was from the U.S. Centers for Disease Control’s National Health and Nutrition Examination Survey (NHANES) accounted for interpreting healthy children [15]. In our center, according to the measurements in 10 children, the coefficient of variation was 0.48% for the lumbar spine. BMD (g/cm2) Z-score less than − 2 for patient’s age and gender was considered as low bone mass for chronological age (LBM).

In growing children, the most important problem in the interpretation of BMD is the effect of bone size on bone mineral content (BMC) and BMD. It means that larger bones due to their greater thickness might have a higher areal BMD. The best solution for this problem is to calculate the bone density per unit volume [17]. To reach this goal, we used bone mineral apparent density (BMAD) to evaluate the child bone density in our study. Estimated volumetric BMAD was calculated for the lumbar spine, using the following formula: 

$$ \mathrm{Lumbar}\ \mathrm{BMAD}=\mathrm{BMC}\ \mathrm{of}\ \mathrm{L}2-\mathrm{L}4/\left(\mathrm{area}\right)\ 1.5 $$

### Statistical analysis

SPSS software version 22 reference [18] was used for Data analysis. Descriptive data are presented as mean ± standard deviation and percentages. Qualitative variables were compared by Chi-square test and Fisher exact test between the case and control groups. Kolmogorov– Smirnov test was used to evaluate the normality of data distribution. Students t-test and Mann-Whitney test were used to compare quantitative variables between two groups. Multiple linear regression model was used to determine independent factors influencing DXA indices. *P* value less than 0.05 was considered to be statistically significant.

## Results

The present study assessed 23 OI patient with an average age of 8.29 ± 4 years and 23 healthy age- and gender-matched children (9 males and 14 females). In OI patients, 60.9% had family history of fracture, 69.6% history of parental consanguinity and 13% had intrauterine fractures. All of the OI children had previous fractures, and 6 had blue sclera. Among all the patients, 6(26%) had type 1 and 17(64%) had type 4 Osteogenesis Imperfecta. In total, 17.4% has taken Pamidronate for more than 3 years and 82.6% less than 3 years.

Table 1 shows the general characteristics of the case and control population. The variables were not normal based on One-Sample Kolmogorov-Smirnov Test (*P* < 0.05), so Mann-Whitney test was used for comparison. Vitamin D level was higher in OI (25.80 ± 15.34) than the control group (18.35 ± 5.35) with *P* value of 0.033. Physical activity was also different between cases and control group (*P* < 0.001). There was no statistically significant difference in other variables, such as age (y), calcium (mg/dl), phosphorous (mg/dl), alkaline phosphates (IU/L), height, weight, BMI, sun exposure, and puberty between the two groups.

**Table 1:** General characteristics of patients and control group and the related comparisons

variable	Control group	OI children	***P***-value
Age (y)	8.29±4	8.29±4	1
Calcium (mg/dl)	9.85±0.49	9.64±0.67	0.230
Phosphorous (mg/dl)	4.42±0.5	4.80±0.83	0.063
Alkaline phosphates (IU/L)	428±74	534±250	0.063
25OHD (ng/ml)	18.35±5.35	25.80±15.34	0.033
Height	124.96±22.10	119±25.10	0.398
Weight	24.43±9.50	24.04±13.20	0.909
BMI	15.24±2.27	15.73±3.50	0.580
BMI Percentile	28.91±6.92	36.52±6.20	0.418
Sun exposure (%)	Low 52%	Low 61%	0.329
	Medium 43%	Medium 26%	
	High 5%	High 13%	
Physical activity (%)	Insufficient 21.7%	Insufficient 73.9%	<0.001
	Sufficient 78.3%	Sufficient 26.1%	
Puberty (%)	Pre pubertal 78%	Pre pubertal 61%	0.359
	Early pubertal 13%	Early pubertal 30%	
	Late pubertal 9%	Late pubertal 9%	
Sex (%)	Male 39.1%	Male 39.1%	0.618
	Female 60.9%	Female 60.9%	

**Fig. 1:**
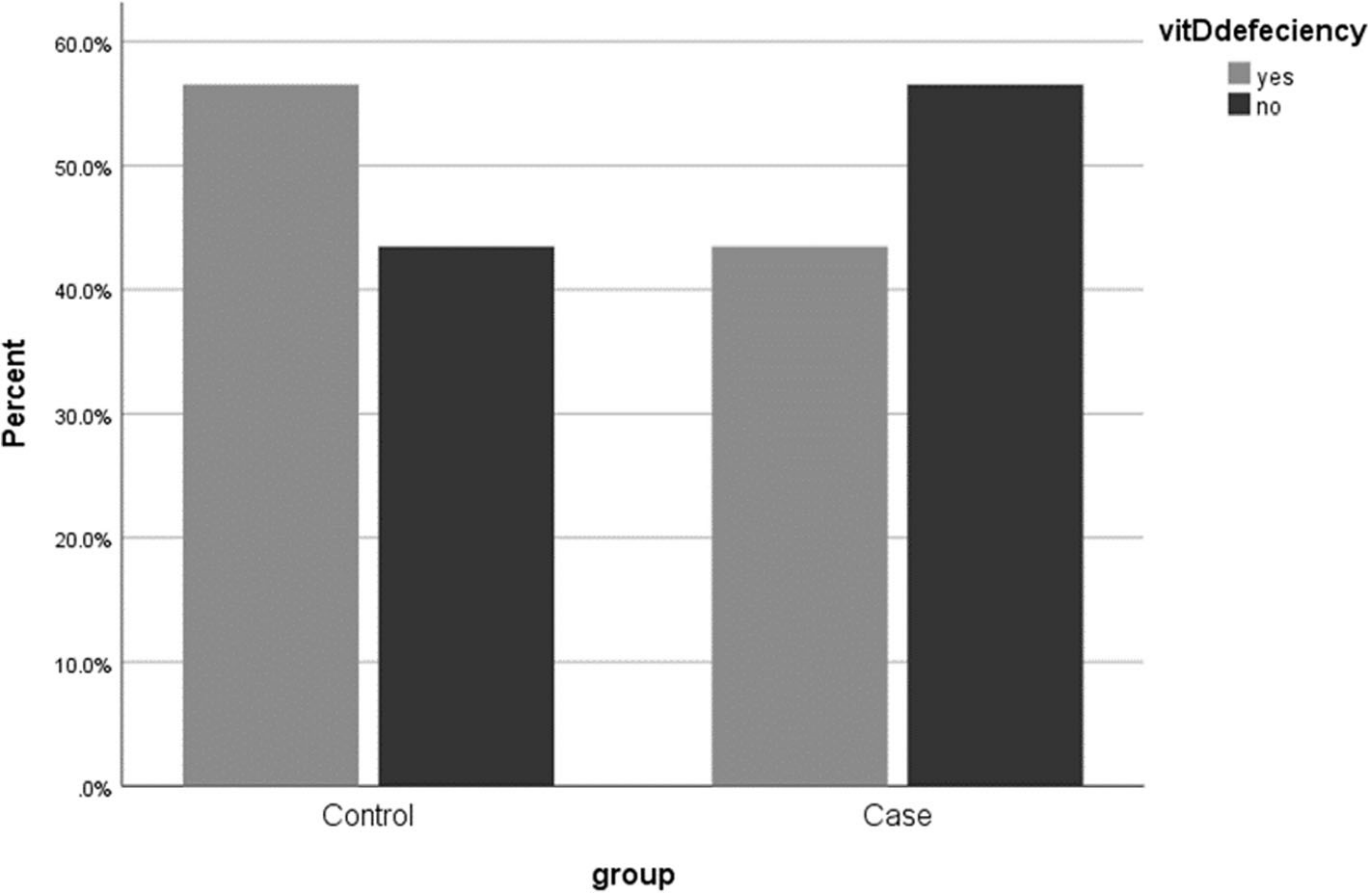
Prevalence of vitamin D defeciency in OI and control group (*P* = 0.376)

About 43% of OI patients and 56% of the control group had vitamin D deficiency, which was not significantly different between patients and controls (*P* = 0.376), Fig. 1. Regression analysis of the associated factors with vitamin D level revealed that none of the below factors, such as age, gender, BMI percentile, sun exposure, puberty, physical activity, intrauterine fracture, courses of Pamidronate injection, Fracture episodes during treatment, Lumbar BMAD and Lumbar z-score can be a predictor of vitamin D level in OI patients (Table 2).

**Table 2:** Results of multiple linear regressions of associated factors with vitamin D level conducted by Enter method

variables	Beta	p-value
age	-0.145	0.803
gender	-0.439	0.117
BMI percentile	0.347	0.291
Puberty	0.870	0.240
Physical activity	0.495	0.147
Sun exposure	-0.852	0820.
Intra uterine fracture	0.087	0.739
courses of Pamidronate injection	-0.575	0.056
fracture episodes during treatment	− 0.198	0.595
Lumbar BMAD	-0.859	0.108
Lumbar z-score	0.820	0.139

None of the participants in the control group (0%), but 13 cases in OI (56%) had low bone mass for chronological age in lumbar area (*P* < 0.001). Lumbar BMD, BMC, BMAD and Z-score were significantly lower in OI (*P* values = 0.002, 0.000, 0.002 and 0.001, respectively). Details of DXA indices of lumbar area in both case and control groups are shown in Table 3. The variables were normal based on One-Sample Kolmogorov-Smirnov Test (*P* > 0.05), so Students t-test were used for comparison However, there were no differences between lumbar low bone mass and variables like: Vitamin D, age, Calcium (mg/dl), Phosphorous (mg/dl), Alkaline phosphates (IU/L), BMI percentile, gender, sun exposure, puberty, physical activity, vitamin D deficiency, intrauterine fracture and fracture episodes during treatment.

**Table 3:** DXA characteristics of lumbar area in both case and control groups

variable	Control group *N* = 23 Mean ± SD	OI children *N* = 23 Mean ± SD	*P*-value	t-value
Lumbar BMD (g/cm2)	0.60 ± 0.15	0.47 ± 0.10	0.002	3.00
Lumbar BMC (g)	25.35 ± 10.74	14.76 ± 5.30	0.000	4.00
Lumbar BMAD (g/cm3)	0.40 ± 0.10	0.31 ± 0.07	0.002	1.00
Lumbar BMD Z-score	-0.53 ± 0.75	-2.27 ± 2.17	0.001	3.00

To evaluate the association between fracture episodes and the possible associated factors such as BMI percentile, intra uterine fracture, Pamidronate therapy initiation time, number of Pamidronate injections, total time of Pamidronate therapy, puberty, vitamin D, age, gender, physical activity, sun exposure, and Lumbar BMAD, multiple linear regression model by Enter method was used.(R Square: 0.914).

Puberty and sun exposure was revalued into 2 groups. Puberty; group1: early puberty and late puberty, group 2: pre puberty. Sun exposure group1: less than 15 min/day and 15–30 min/day, group 2: more than 30 min.

Fracture episodes during treatment was significantly associated with the age, gender, initiation time of Pamidronate therapy, number of Pamidronate injection, puberty and sun exposure (*P* values = 0.003, 0.015, 0.000, 0.002, 0.005 and 0.006, respectively). Table 4 shows the association between fracture episodes during treatment and some possible associated factors in OI children. In addition, it was shown that number of fracture episodes had raised in patients who had received Pamidronate for more than 3 years compared with those received below 3 years (*P* values = 0.047).

**Table 4:** Results of multiple linear regressions of associated factors with fracture episodes during treatment conducted by Enter method. R Square: 0.914

variables	Std.Error	Beta	*p*-value
age	0.197	1.314	**0.003**
gender	0.572	-0.349	**0.015**
BMI percentile	0.010	-0.075	0.548
Puberty	1.128	0.850	**0.005**
Physical activity	0.683	0.113	0.402
Sun exposure	0.760	-0.549	**0.006**
Vitamin D	0.020	-0.108	0.421
Intra uterine fracture	0.837	-0.266	0.053
Pamidronate therapy initiation time	0.249	-2.462	**0.000**
Number of Pamidronate injection	0.058	-0.878	**0.002**
Total time of Pamidronate therapy	0.264	-0.603	0.100
Lumbar BMAD	14.062	-0.075	0.511

## Discussion

The present study showed that children with Osteogenesis Imperfecta had higher mean vitamin D level than the healthy control group, which might have been due to supplementary calcium and vitamin D consumption. In spite of having higher vitamin D levels found in patients with OI, 43.4% of them had criteria of vitamin D deficiency, which can reflect upon supplementary vitamin D dosage insufficiency, poor compliance or secondary causes. We showed that 56.5% of the control group had vitamin D deficiency. A previous study in south of Iran showed that 81.3% of healthy children had vitamin D deficiency, which was due to insufficient sun exposure, inadequate physical activity, pubertal stage and advancing age [16]. To the best of our knowledge, our study is amongst the few that reported the prevalence of vitamin D deficiency in OI pediatric patients in the Middle East. Most of the previous studies that had assessed vitamin D level in OI patients, were from North America [6, 9, 11, 12, 19] and Brazil [20]. The present study was similar to a report from Edouard et al. in 2011 which showed that 52% of 71 OI patients in Montreal Canada had vitamin D deficiency [19]. In addition, Zambrano et al. in 2016 revealed that 35.5% and 51.9% of 52 Brazilian OI children had vitamin D deficiency and insufficiency, respectively [20]. In line with our findings, Edouard et al. in 2011 reported that there was no association between 25OHD level with measures of bone mineralization in OI children [19]. There are considerable evidences that vitamin D deficiency raises the risk of falls and fractures [15]. Consequently, regular evaluation of vitamin D deficiency and if so, its treatment has great benefit in OI patients who suffer from frequent disabling fractures.

Most of children with OI, show short stature with small body size for age as a result of growth retardation. Hence, we used volumetric BMD or BMAD for a more accurate interpretation of BMD results, due to the effect of body size on BMD [17]. BMD parameters like Lumbar BMD, BMAD and Z-score were very low in our cases in comparison with control group, which was anticipated in a population with OI. There was no significant association between Lumbar BMAD and Z-score and vitamin D level, which is reflection of BMD parameters of OI patients that varies in the severity of disease. Bisphosphonate treatment is established as a standard treatment for moderate to severe cases, who suffer from bone fractures. Bisphosphonates not only increase lumbar spine (LS) BMD but also improve bone structures, increase mobility and reduce fracture risk [4, 6]. In 2019 Bains et al. in a cohort multicenter study on 299 individuals with OI type I (a mild form of disease), revealed that people who were receiving bisphosphonates, BMD and mobility had increased, fracture rate and scoliosis was decreased in comparison with the group who did not took bisphosphonates [21]. There is no unanimous view as to when bisphosphonate treatment should be initiated for individuals with OI [4]. Our results provide evidence that time of treatment initiation and courses of Pamidronate injection is linked with fracture rates, which means that early start of treatment and regular intervals might reduce fracture rates. In 2008, Poyrazoglu et al. revealed that younger children with OI responded faster to Pamidronate treatment by increase in their BMD Z-score [8]. Fracture rates was in correlation with puberty and sun exposure. Another finding of our study was that Pamidronate therapy duration of more than 3 years (long term treatment) increased fracture rate without any change in BMD parameters, which might lead to bone fragility. In contrast, one study on 79 Swedish children reported that after more than 4 years of Pamidronate treatment, fracture rate was reduced [4].

Our study has several strengths. It is amongst limited case-control studies, assessing vitamin D status, BMD and its associated factors in children with OI as a rare disease in the Middle East. Also, we used volumetric BMD (BMAD) in OI children to reduce the effect of bone size on BMC and BMD, even though there are limited reports. Despite these strengths, we had several limitations. Although we used a standard questionnaire, physical activity, sun exposure and fracture data were self-reported and underwent recall bias. Second, all the patients had received vitamin D supplements since the time of diagnosis, so we could not evaluate the true prevalence of vitamin D deficiency, however, they had vitamin D deficiency in spite of receiving supplements. Last but not the least, was our mandatory small sample size, which could have influenced the results e.g. regression analysis. Multicenter studies are warranted to find all the correlates.

## Conclusions

This study showed that 43.4% of OI children in southern Iran had vitamin D deficiency. There was no association between Vitamin D level and BMD parameters. In total, 56% of OI children had low bone mass for chronological age in lumbar area. Our results provide evidence that early initiation of Pamidronate therapy and courses of Pamidronate injections led to reduction in fracture rates. However, treatment period of more than 3 years had adverse effect on fracture rates. Performing DXA before treatment and monitoring it during treatment is recommended in children with OI.

## Data Availability

The datasets used and/or analyzed during the current study are available from the corresponding author on reasonable request.

## Abbreviations

25OHD: 25-hydroxyvitamin D
ALP: Alkaline phosphatase
BMI: Body mass index
BMD: Bone mineral density
BMAD: bone mineral apparent density
Ca: Calcium
DXA: Dual-energy X-ray absorptiometry
LBM: low bone mass for chronological age
NHANES: National Health and Nutrition Examination Survey
OI: Osteogenesis imperfecta
P: Phosphorous
SUMS: Shiraz University of Medical Sciences
SPSS: SPSS statistical software
